# Correlation between miRNA target site polymorphisms in the 3′ UTR of *AVPR1A* and the risk of hypertension in the Chinese Han population

**DOI:** 10.1042/BSR20182232

**Published:** 2019-05-14

**Authors:** Liuping Zhang, Jinwei Liu, Peng Cheng, Fangchao Lv

**Affiliations:** 1 Department of Cardiology, Zhuji People’s Hospital of Zhejiang Province, No. 9 Jianmin Road, Tao Zhu street, Zhuji, Zhejiang, China; 2 Department of Emergency, Yidu Central Hospital of Weifang, Qingzhou, Shangdong Province, China; 3 Department of Intensive Care Medicine, Yidu Central Hospital of Weifang, Qingzhou, Shangdong Province, China; 4 Department of Cardiology, Zhejiang hospital, No.12 Ling yin Road,Hangzhou, Zhejiang, China

**Keywords:** Arginine vasopressin receptor 1a, hypertension, microRNA, single nucleotide polymorphism

## Abstract

We aimed to study the relationship between rs11174811 and rs3803107 single nucleotide polymorphisms (SNPs) in miRNA target sites of the 3′ UTR in the arginine vasopressin receptor 1a gene (*AVPR1A*) and the risk of hypertension in the Chinese Han population. The genotypes at rs11174811 and rs3803107 were analyzed by direct sequencing in 425 Chinese Han patients with hypertension and 425 healthy subjects. AVPR1A expression was investigated by transfecting miR-526b, miR-375, and miR-186 mimics into human umbilical vein endothelial cells (HUVECs) containing *AVPR1A* rs11174811 CC, CA/AA and *AVPR1A* rs3803107 GG, GA/AA genotypes. The A alleles of rs11174811 (adjusted OR = 1.424, 95% CI: 1.231–1.599, *P*<0.001) and rs3803107 (adjusted OR = 1.222, 95% CI: 1.092–1.355; *P*=0.001) were high risk factors for hypertension. Plasma levels of miR-526b, miR-375, and miR-186 were higher in the study group than in the control group (*P*<0.001). The expression levels of *AVPR1A* mRNA in AVPR1A rs11174811 and rs3803107 mutant HUVECs were higher than those in wild-type cells (*t* = 8.811, 4.068 and *P*=0.001, 0.015, respectively). The single nucleotide polymorphisms rs11174811 and rs3803107 in the *AVPR1A* gene are associated with an increased risk of hypertension in the Chinese Han population. This may be related to the effect of these variants on the regulation of *AVPR1A* expression by miRNAs.

## Introduction

Essential hypertension is a common cardiovascular disease in middle-aged and elderly populations. Its clinical characteristics are elevated blood pressure and multiple cardiovascular crises, which seriously affect the physical and mental health of patients and can lead to numerous complications and death [1].

Arginine vasopressin (AVP) is a vasoconstricting neuroendocrine hormone secreted by the supraventricular nucleus and paraventricular ventricles. AVP interacts with its receptor, AVPR1A, to cause the contraction of peripheral blood vessels [2,3]. Studies have shown that AVP levels are significantly elevated in the plasma of patients with heart failure and AVP can induce myocardial fibroblast proliferation and promote myocardial fibrosis through its V1a receptor [4,5].

The *AVPR1A* gene, encoding a receptor for AVP, is located at 12q14.2. It belongs to a subfamily of G protein-coupled receptors that also includes, AVPR1B, V2R, and oxytocin receptor [2]. AVPR1A activity is mediated by G proteins, which stimulate the phosphatidylinositol-calcium second messenger system [6]. The receptor affects cell contraction and proliferation, platelet aggregation, the release of coagulation factors, and glycogenolysis [7]. *AVPR1A* gene polymorphisms have become a hot research topic in recent years. The rs11174811 single nucleotide polymorphism (SNP) in the 3′ UTR of *AVPR1A* has been associated with increased arterial blood pressure [8]. Variation at the rs3803107 locus of the *AVPR1A* gene is more common in the Chinese Han population than other populations, with a minimum allele frequency (MAF) as high as 0.1650 (Table 1). Both rs11174811 and rs3803107 are located in the 3′ UTR region of *AVPR1A*, in the target sites for miRNAs. TargetScan 3.1 analysis predicted that the miR-526b target region was located at the rs11174811 locus, while the miR-375 and miR-186 target regions were located at the rs3803107 locus.

**Table 1:** *AVPR1A* 3′ UTR SNP site information

SNP	Chromosome	MAF (%)	miRNAs
rs11174811	12:63146696	0.0485 (A)	miR-526b
rs3803107	12:63147054	0.1650 (A)	miR-375; miR-186

In the present study, we analyzed the relationship between the SNPs, rs11174811, and rs3803107 in miRNA target sites within the *AVPR1A* 3′ UTR, and the risk of hypertension in the Chinese Han population. The role of miRNAs in regulating *AVPR1A* expression was also investigated.

## Materials and methods

### Subjects

A total of 425 patients with ER, who were admitted to Zhuji People’s Hospital of Zhejiang Province and Yidu Central Hospital of Weifang from June 2015 to December 2017, were randomly selected. This group of patients included 276 males and 149 females, ranging in age from 42–85 years, with an average age of 59.28 ± 7.84 years. The diagnostic criteria were: sitting systolic blood pressure (SBP) ≥140 mmHg and/or diastolic blood pressure (DBP) ≥90 mmHg [9]. Patients were excluded from the study if they had secondary hypertension, diabetes, coronary heart disease, valvular heart disease, and other organic heart disease, cancer, or immune system diseases. A control group of 425 healthy subjects was recruited, with matching age and gender to those of the patients in the study group. Control subjects ranged in age from 41–85 years, with a mean age of 60.82 ± 13.45 years. Subjects were included in the control group if they met the following criteria: SBP <140 mmHg; DBP <90 mmHg; and the absence of diabetes, coronary heart disease, organic heart disease, cancer, and immune system diseases. The present study was approved by the Medical Ethics Committee of Zhuji People’s Hospital of Zhejiang Province and Yidu Central Hospital of Weifang and all subjects gave informed consent.

### Genotyping

**Figure 1:**
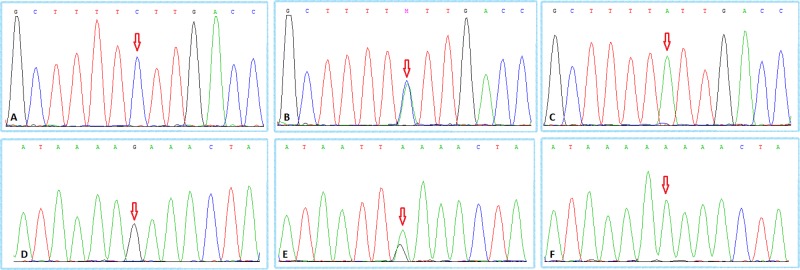
Sanger sequencing results (**A**) *AVPR1A* rs11174811 locus CC genotype; (**B**) *AVPR1A* rs11174811 locus CA genotype; (**C**) *AVPR1A* rs11174811 locus AA genotype; (**D**) *AVPR1A* rs3803107 locus GG genotype; (**E**) *AVPR1A* rs3803107 locus GA genotype; (**F**) *AVPR1A* rs3803107 locus AA genotype. The red arrow indicates the mutated nucleotide site.

Venous blood (5 ml) was collected from all subjects, plasma cells were separated, and genomic DNA was extracted using a QIAamp DNA Blood Mini Kit (Qiagen, Venlo, Netherlands). Genotypes at the rs11174811 and rs3803107 loci in the *AVPR1A* gene’s 3′ UTR were detected using Sanger sequencing technology. Sequencing primers for rs11174811 (F: 5′-AGG CCA CTG CCA GTT GTA AA-3′; R: 5′-GAG TAC AAG TGC CTG GGG TG-3′) and rs3803107 (F: 5′-AGT GCC GCA TTT TAT GTG ACT-3′; R: 5′-ATG CAG GTC TGA TTC CCA GA-3′) were designed based on the published chromosomal location of these SNPs (Table 1). Representative sequencing results for each genotype are shown in Figure 1.

### miRNA expression analysis

Plasma miRNAs were extracted using a PureLink RNA Mini Kit™ (catalog number 12183018A, Invitrogen, Carlsbad, CA, U.S.A.). miRNA was reverse transcribed into cDNA using a reverse transcription kit (TOYOBO, Osaka, Japan) and reverse transcription primers were purchased from Guangzhou Ribo Bio Co. Ltd (Guangzhou, China). U6 RNA was used as a miRNA control. Quantitative real-time PCR was performed using a SYBR-Green PCR kit (catalog number 208054, TOYOBO), with three replicate wells per sample. miRNA expression level was analyzed using the 2^−ΔΔ*C*^_T_ method.

### Cell culture and transfection

Human umbilical vein endothelial cells (HUVECs; PCS-100-013TM; ATCC, Manassas, VA, U.S.A.) with rs11174811 CC and CA/AA genotypes and HUVECs with rs3803107 GG and GA/AA genotypes were constructed using CRISPR/Cas9 technology, which was provided by BioGene Cas9 Inc. (Chongqing, China). HUVECs were cultured in M199 medium containing 10% fetal bovine serum (Cambrex Biosciences, Walkersville, MD, U.S.A.), 10 mM HEPES (Sigma–Aldrich, St. Louis, MO, U.S.A.), and 1% penicillin/streptomycin. Cells were cultured for 2–6 passages at 37°C and 5% CO_2_. HUVECs (1.5 × 10^5^ cells) containing rs11174811 CC and CA/AA genotypes were transfected with 100 ng of miRNA-526b mimic. Total 100 ng of miRNA-375 mimic and miRNA-186 mimic was transfected into 1.5 × 10^5^ HUVECs containing rs3803107 GG and GA/AA genotypes, respectively. The ssEGFP miRNA (100 ng) was transfected into control cells. AVPR1A protein expression was analyzed 48 h after transfection.

### Quantitative real-time PCR

Total RNA was extracted from collected cells using an RNeasy Mini Kit (Qiagen) and reverse transcribed into cDNA using a reverse transcription kit (TOYOBO). *AVPR1A* was amplified using the following primers: forward, 5′-CTTTTGTGATCGTGACGGCTTA-3′ and reverse, 5′-TGATGGTAGGGTTTTCCGATTC-3′. GAPDH was used as an internal reference and amplified using the following primers: forward, 5′-ACCCAGAAGACTGTGGATGG-3′ and reverse, 5′-TTCTAGACGGCAGGTCAGGT-3′. qRT-PCR was performed using a SYBR-Green PCR kit (TOYOBO), with three replicate wells per sample. miRNA expression level was analyzed using the 2^−ΔΔ*C*^_T_ method.

The reaction mixture comprised 10 μl of SYBR Green Real Master Mix (Promega), 1.6 μl of primer mix (200 nM), and 1.6 μl of cDNA template. The thermal cycling conditions were as follows: 3 min at 95°C for denaturation, then 38 cycles of 95°C for 10 s, 60°C for 30 s, and 72°C for 30 s. After the PCR was completed, a melting curve analysis was performed to ensure the purity of the PCR product.

### Western blotting

Total protein was extracted from collected cells using a membrane and cytosolic protein extraction kit (no. P0013B; Beyotime, Jiangsu, China) and AVPR1A protein expression was detected by western blotting (anti-AVPR1A polyclonal antibody, Cat# 720364; ThermoFisher, Waltham, MA, U.S.A.). After calibration by BCA protein assay kit (23225, Pierce Pharmaceuticals, Melbourne, VIC, Australia), the supernatant was denatured in 4× Lemmli sample buffer (161-0747, Bio-Rad) solution at 95°C for 10 min. Protein was isolated by Bio-Rad device and transferred to PVDF membrane (ISEQ00010, Merck Millipore, Darmstadt, Germany). Then the PVDF membrane was incubated in 5% skim milk at room temperature for 1 h and incubated overnight with the primary antibody (AVP:1:1000, AB1565, Merck Millipore; AVPR1a:1:200, sc-18096, Santa Cruz, CA, U.S.A.; AVPR1b:1:10000, PAB11503, Abnova, Taipei, Taiwan; glucocorticoid receptor (GR): 1:200, AB 2768, Cambridge, U.K.). After washing in a buffer (TBS-0.1% Tween 20), the membrane was incubated with the second antibody (1:10000) coupled with horseradish peroxidase (HRP) for 2 h at 4°C. The target protein signal was detected by ECL detection kit (Immobilon western chemiluminescent HRP substrate p90720, Merck Millipore), and the PVDF membranes were photographed using Image Quant LAS 4000 Mini (GE Healthcare, Buckinghamshire, U.K.). Quantity One software (Bio-Rad, Hercules, CA, U.S.A.) was used to quantitate signals. After standardization using glyceraldehyde-3-phosphate dehydrogenase (GAPDH, ab181602, Abcam) as the internal control, the signal intensity was expressed in any unit of optical density.

### Statistical analysis

Continuous variables are expressed as mean ± S.D. (x ± s) and comparisons between groups were performed using an independent sample Student’s *t* test. Categorical variables are expressed as a percentage (n[%]) and comparisons between groups were performed using a χ^2^ test. A χ^2^ test was also used to determine whether genotype frequencies were consistent with those at Hardy-Weinberg equilibrium (HWE). The association of SNPs with hypertension risk was determined based on the distribution of allele frequencies and genetic models (dominant and recessive models). Odds ratios (OR) and 95% CI were used in unconditional logistic regression analysis, with corrections for age, sex, body mass index (BMI), smoking, drinking, and family history of hypertension. Statistical analysis was performed using SPSS21.0 software (IBM, Chicago, IL, U.S.A.). All tests were two-tailed and differences were considered statistically significant at *P*<0.05.

## Results

### Demographic characteristics

There were no significant differences in age or gender between the study group and the control group (*P*>0.05). BMI, smoking, drinking, and a family history of hypertension were all more frequent in the study group than in the control group. DBP and SBP were also significantly higher in the study group than in the control group (*P*<0.05, Table 2).

**Table 2:** General data

Characteristic	Case (*n*=425)	Control (*n*=425)	*P*
Age (mean ± S.D.)	59.28 ± 7.84	60.82 ± 13.45	0.224
Gender (n[%])			0.668
Male	276 (64.94%)	270 (63.53%)	
Female	149 (35.06%)	155 (36.47%)	
BMI (kg/m^2^, mean ± S.D.)	25.30 ± 2.77	23.38 ± 2.36	<0.001
Smoking [n(%)]			<0.001
Yes	179 (42.12%)	115 (27.06%)	
No	246 (57.88%)	310 (72.94%)	
Drinking [n(%)]			0.007
Yes	194 (45.65%)	155 (36.47%)	
No	231 (54.35%)	270 (63.53%)	
DBP (mmHg, mean ± S.D.)	83.22 ± 9.49	74.20 ± 12.37	<0.001
SBP (mmHg, mean ± S.D.)	143.98 ± 17.10	121.11 ± 17.28	<0.001
Family history [n(%)]			<0.001
Yes	123 (28.94%)	63 (14.82%)	
No	302 (71.06%)	362 (85.18%)	

The criterion for smoking was: smoking more than five cigarettes a week. The criterion for drinking was: consumption of alcohol more than three times a year, in excess of 50 mL each time.

### *AVPR1A* 3′ UTR SNPs and risk of hypertension

The genotype distributions of the rs11174811 and rs3803107 loci were consistent with their frequencies at HWE (*P*=0.252, 0.274, respectively). Using the wild type (CC) of the rs11174811 locus as a reference, the risk of hypertension was increased in homozygous, dominant, and recessive models (adjusted OR = 1.927, 95% CI: 1.574–2.046, *P*<0.001; adjusted OR = 1.261, 95% CI: 1.032–1.485, *P*=0.024; adjusted OR = 1.935, 95% CI: 1.581–2.053, *P*<0.001; respectively). Heterozygous (CA) models were without risk of hypertension (adjusted OR = 0.953, 95% CI: 0.697–1.229, *P*=0.819). Using the C allele as a reference, the A allele was a high risk factor for hypertension (adjusted OR = 1.424, 95% CI: 1.231–1.599, *P*<0.001; Table 3). Taking the GG genotype of the rs3803107 locus as a reference, the risk of hypertension in the homozygous and recessive models was significantly increased (adjusted OR = 1.583, 95% CI: 1.288–1.823, *P*<0.001; adjusted OR = 1.570, 95% CI: 1.283–1.798, *P*<0.001; respectively). Heterozygous and dominant models did not have an increased risk of hypertension (adjusted OR = 1.031, 95% CI: 0.868–1.210, *P*=0.772; adjusted OR = 1.151, 95% CI: 0.995–1.320, *P*=0.058; respectively). With the G allele as a reference, the A allele was a high risk factor for hypertension (adjusted OR = 1.222, 95% CI: 1.092–1.355, *P*=0.001; Table 3)

**Table 3:** *AVPR1A* 3′ UTR SNP genotypes and allele frequencies

	Case (*n=* 425)	Control (*n=* 425)	HWE *P*	Adjusted OR (95% CI)	*P*
**rs11174811**					
CC	365 (85.88%)	387 (91.06%)	0.252	1.000 (reference)	
CA	31 (7.29%)	36 (8.47%)		0.953 (0.697–1.229)	0.819
AA	29 (6.82%)	2 (0.47%)		1.927 (1.574–2.046)	<0.001
Dominant model				1.261 (1.032–1.485)	0.024
Recessive model				1.935 (1.581–2.053)	<0.001
C	761(89.53%)	810 (95.29%)		1.000 (reference)	
A	89 (10.47%)	40 (4.71%)		1.424 (1.231–1.599)	<0.001
**rs3803107**					
GG	271 (63.76%)	298 (70.12%)	0.274	1.000 (reference)	
GA	108 (25.41%)	112 (26.35%)		1.031 (0.868–1.210)	0.772
AA	46 (10.82%)	15 (3.53%)		1.583 (1.288–1.823)	<0.001
Dominant model				1.151 (0.995–1.320)	0.058
Recessive model				1.570 (1.283–1.798)	<0.001
G	650 (76.47%)	708 (83.29%)		1.000 (reference)	
A	200 (23.53%)	142 (16.71%)		1.222 (1.092–1.355)	0.001

### Stratified analysis of *AVPR1A* 3′ UTR SNPs and risk factors for hypertension

The stratified analysis of age, sex, BMI, smoking status, drinking status, and family history of hypertension showed that the risk of hypertension was increased in subjects with the *AVPR1A* rs11174811 mutation (CA/AA) if they also had an age >60 years (adjusted OR = 1.401; 95% CI: 1.011–1.810; *P*=0.043), BMI <24 kg/m^2^ (adjusted OR = 1.569; 95% CI: 1.037–2.184; *P*=0.033), or family history of hypertension (adjusted OR = 1.367; 95% CI: 1.052–1.567; *P=*0.021; Table 4). The risk of hypertension was significantly increased in subjects with the *AVPR1A* rs3803107 mutation (GA/AA) only if they had a family history of hypertension (adjusted OR = 1.262; 95% CI: 1.006–1.514; *P*=0.044; Table 5).

**Table 4:** Stratified analysis of the correlation between rs11174811 genotype and general data

Characteristic	Case (*n*=425)	Control (*n*=425)	Adjusted OR (95% CI)	*P*
Age (years)				
<60				
CC	240 (56.47%)	204 (48.00%)	1.000 (reference)	
CA/AA	31 (7.29%)	16 (3.76%)	1.220 (0.923–1.486)	0.160
≥ 60				
CC	125 (29.41%)	183 (43.06%)	1.000 (reference)	
CA/AA	29 (6.82%)	22 (5.18%)	1.401 (1.011–1.810)	0.043
Gender				
Male				
CC	235 (55.29%)	243 (57.18%)	1.000 (reference)	
CA/AA	41 (9.65%)	27 (6.35%)	1.226 (0.953–1.196)	0.112
Female				
CC	130 (30.59%)	144 (33.88%)	1.000 (reference)	
CA/AA	19 (4.47%)	11 (2.59%)	1.335 (0.905–1.729)	0.144
BMI (kg/m2)				
< 24				
CC	108 (25.41%)	255 (60.00%)	1.000 (reference)	
CA/AA	21 (4.94%)	24 (5.65%)	1.569 (1.037–2.184)	0.033
≥ 24				
CC	257 (60.47%)	132 (31.06%)	1.000 (reference)	
CA/AA	39 (9.18%)	14 (3.29%)	1.114 (0.889–1.300)	0.349
Smoking				
Yes				
CC	151 (35.53%)	101 (23.76%)	1.000 (reference)	
CA/AA	28 (6.59%)	14 (3.29%)	1.113 (0.826–1.375)	0.510
No				
CC	214 (50.35%)	286 (67.29%)	1.000 (reference)	
CA/AA	32 (7.53%)	24 (5.65%)	1.335 (0.992–1.679)	0.056
Drinking				
Yes				
CC	164 (38.59%)	141 (33.18%)	1.000 (reference)	
CA/AA	30 (7.06%)	14 (3.29%)	1.268 (0.953–1.549)	0.102
No				
CC	201 (47.29%)	246 (57.88%)	1.000 (reference)	
CA/AA	30 (7.06%)	24 (5.65%)	1.235 (0.905–1.572)	0.184
Family history				
Yes				
CC	95 (22.35%)	58 (13.65%)	1.000 (reference)	
CA/AA	28 (6.59%)	5 (1.18%)	1.367 (1.052–1.567)	0.021
No				
CC	270 (63.53%)	329 (77.41%)	1.000 (reference)	
CA/AA	32 (7.53%)	33 (7.76%)	1.092 (0.804–1.400)	0.612

The criterion for smoking was: smoking more than five cigarettes a week. The criterion for drinking was: consumption of alcohol more than three times a year, in excess of 50 ml each time.

**Table 5:** Stratified analysis of the correlation between rs3803107 genotype and general data

Characteristic	Case (*n=* 425)	Control (*n=* 425)	Adjusted OR (95% CI)	*P*
Age (years)				
<60				
GG	169 (39.76%)	151 (35.53%)	1.000 (reference)	
GA/AA	102 (24.00%)	69 (16.24%)	1.129 (0.949–1.328)	0.175
≥60				
GG	102 (24.00%)	147 (34.59%)	1.000 (reference)	
GA/AA	52 (12.24%)	58 (13.65%)	1.154 (0.880–1.482)	0.318
Gender				
Male				
GG	183 (43.06%)	190 (44.71%)	1.000 (reference)	
GA/AA	93 (21.88%)	80 (18.82%)	1.096 (0.909–1.304)	0.353
Female				
GG	88 (20.71%)	108 (25.41%)	1.000 (reference)	
GA/AA	61 (14.35%)	47 (11.06%)	1.258 (0.982–1.582)	0.070
BMI (kg/m^2^)				
<24				
GG	84 (19.76%)	197 (46.35%)	1.000 (reference)	
GA/AA	45 (10.59%)	82 (19.29%)	1.185 (0.861–1.605)	0.318
≥24				
GG	187 (44.00%)	101 (23.76%)	1.000 (reference)	
GA/AA	109 (25.65%)	45 (10.59%)	1.090 (0.943–1.243)	0.254
Smoking				
Yes				
GG	113 (26.59%)	80 (18.82%)	1.000 (reference)	
GA/AA	66 (15.53%)	35 (8.24%)	1.116 (0.908–1.342)	0.313
No				
GG	158 (37.18%)	218 (51.29%)	1.000 (reference)	
GA/AA	88 (20.71%)	92 (21.65%)	1.163 (0.948–1.411)	0.151
Drinking				
Yes				
GG	117 (27.53%)	108 (25.41%)	1.000 (reference)	
GA/AA	77 (18.12%)	47 (11.06%)	1.194 (0.975–1.439)	0.088
No				
GG	154 (36.24%)	190 (44.71%)	1.000 (reference)	
GA/AA	77 (18.12%)	80 (18.82%)	1.096 (0.884–1.338)	0.427
Family history				
Yes				
GG	74 (17.41%)	48 (11.29%)	1.000 (reference)	
GA/AA	49 (11.53%)	15 (3.53%)	1.262 (1.006–1.514)	0.044
No				
GG	197 (46.35%)	250 (58.82%)	1.000 (reference)	
GA/AA	105 (24.71%)	112 (26.35%)	1.098 (0.913–1.307)	0.335

The criterion for smoking was: smoking more than five cigarettes a week. The criterion for drinking was: consumption of alcohol more than three times a year, in excess of 50 ml each time.

### Plasma miRNA expression levels

**Figure 2:**
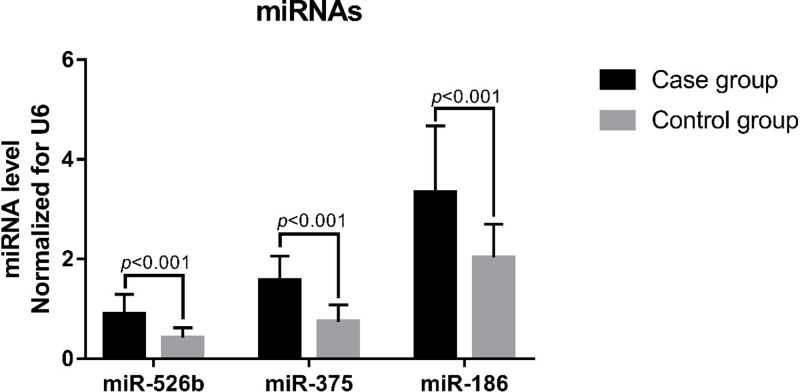
Plasma miRNA levels. Total 425 subjects each in the study and control groups

Plasma levels of miR-526b, miR-375, and miR-186 were significantly higher in the study group than in the control group (*P*<0.001, Figure 2).

**Figure 3:**
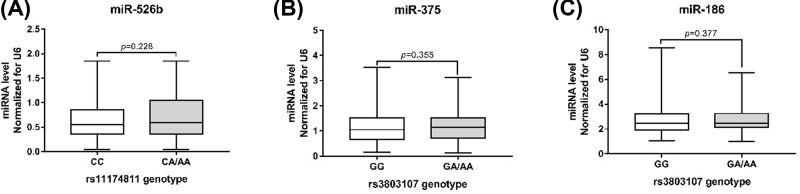
Correlation between *AVPR1A* 3′ UTR SNPs and plasma miRNA levels (**A**) rs11174811 locus genotype and plasma miR-526b levels, 725 cases with CC genotype and 98 cases with CA/AA; (**B**) rs3803107 locus genotype and plasma miR-375 levels, 569 cases with GG genotype and 281 cases with CA/AA; (**C**) rs3803107 locus genotype and plasma miR-186 levels, 569 cases with GG genotype and 281 cases with CA/AA.

There was no difference in plasma miRNA-526b levels between subjects with wild type (CC) or mutant (CA/AA) genotypes at the rs11174811 locus (*P*=0.228, Figure 3A). Likewise, there was no difference in plasma miR-375 and miR-186 levels between subjects with wild-type (GG) or mutant (GA/AA) genotypes at the rs3803107 locus (*P*=0.355 and 0.377, respectively; Figure 3B,C).

### The correlation between *AVPR1A* 3′ UTR SNPs and plasma miRNA levels

**Figure 4:**
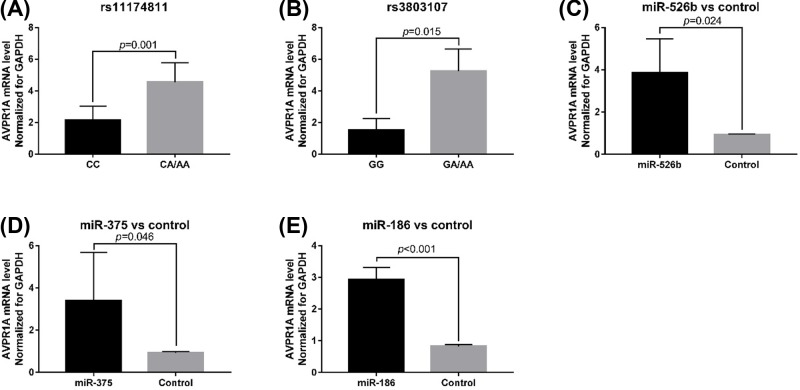
*AVPR1A* mRNA expression according to rs11174811 and rs3803107 genotypes (**A**) *AVPR1A* mRNA expression level in cells with different genotypes at rs11174811 locus. (**B**) *AVPR1A* mRNA expression level in cells with different genotype at rs3803107 locus. (**C**) The expression level of *AVPR1A* in cells transfected with miRNA-526b and control; (**D**) the expression level of *AVPR1A* in cells transfected with miRNA-375 and control; (**E**) the expression level of *AVPR1A* in the cells transfected with miRNA-186 and control.

**Figure 5:**
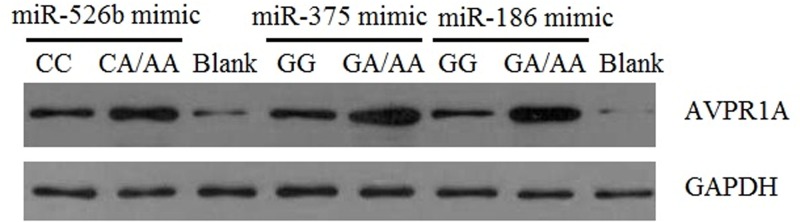
AVPR1A protein expression by western blotting The loading quantity of protein sample was 30 µg. Blank indicates that the cells were transfected with 100 ng ssEGFP miRNA. Lane 1: *AVPR1A* rs11174811 CCVE type HUVEC line, lane 2: *AVPR1A* rs11174811 CA/AA genotype HUVEC line, lane 4 and 6: *AVPR1A* rs3803107 GG genotype HUVECs. Lane 5 and 7: *AVPR1A* rs3803107 GA/AA genotype of HUVEC line.

The effect of the rs11174811 and rs3803107 genotypes on *AVPR1A* mRNA expression was investigated. *AVPR1A* mRNA expression level was higher in cells with mutant genotypes at rs11174811 (*t* = 8.811, *P*=0.001) and rs3803107 (*t* = 4.068; *P*=0.015) loci than in wild-type cells (Figure 4A,B). *AVPR1A* mRNA expression increased in HUVECs after transfection with miR-526b, miR-375, and miR-186 (*P*<0.05, Figure 4C–E). Western blotting showed that AVPR1A protein expression level was also higher in mutant cells than in wild-type cells (Figure 5).

## Discussion

In the present study, we analyzed the correlation between rs11174811 and rs3803107 SNPs in the 3′ UTR of the *AVPR1A* gene and the risk of hypertension in the Chinese Han population. Our results showed that carriers of the A allele at the rs11174811 locus had a 1.424 times higher risk of developing hypertension than carriers of the C allele (95% CI: 1.231–1.599, *P*<0.001). The rs3803107 locus A allele was associated with a 1.222 times higher risk of hypertension than the G allele (95% CI: 1.092–1.355, *P*=0.001). Furthermore, rs11174811 and rs3803107 mutant *AVPR1A* were expressed at a higher level than wild-type *AVPR1A* in HUVECs. Therefore, we believe that these SNPs in the 3′ UTR of the *AVPR1A* gene affect miRNA-mediated regulation of *AVPR1A* gene expression.

As the population ages, the incidence of hypertension in China is gradually increasing. Hypertension is an independent risk factor for cardiovascular and cerebrovascular diseases, which are important causes of mortality and disability in the elderly. Therefore, the prevention and treatment of hypertension in the elderly are especially important [10,11]. The development of hypertension is caused by multiple factors, including the interaction of genetic and environmental factors [12,13]. During the development of hypertension, the renin-angiotensin-aldosterone system, the sympathetic nervous system, and ion transporters in the kidneys and other tissues play important roles [14–16]

In recent years, a correlation between non-coding RNAs (ncRNAs) and human diseases has been reported. There have been many studies on the relationship between ncRNAs and cardiovascular diseases or hypertension and numerous miRNAs have been shown to have an important role in the process of hypertension [17–19]. Studies have found that the rs11174811 SNP in an miRNA binding site within the *AVPR1A* gene, and is correlated with elevated blood pressure and risk of myocardial infarction in Caucasians [20]. In addition, the researchers found that this SNP was not correlated with elevated blood pressure in a southern Indian population [8]. This suggests that the correlation between rs11174811 and hypertension risk may be related to ethnicity. The subjects in the present study were Han Chinese from southern China. A total of 425 hypertensive patients were included, which is a relatively small number. We found that the MAF (A) at the rs11174811 locus was 4.71%, while data from the 1000 Genomes Project database showed that the frequency of this allele was 4.85% in the Han population from Beijing, China and 2.86% in the Han population from southern China. This indicates that the subjects selected in the present study are representative of the population and the results are highly reliable. The rs3803107 locus is also located in the 3′ UTR region of *AVPR1A*, but few studies have focussed on this site. Tansey et al. [21] studied four SNPs in *AVPR1A* (rs3803107, rs1042615, rs3741865, and rs11174815) and three microsatellites (RS3, RS1, and AVR) to assess their association with autism in an Irish population. They found that, out of these four SNPs, only rs11174815 was correlated with autism. The results of the present study showed that rs3803107 was correlated with hypertension risk in a Chinese Han population.

rs11174815 and rs3803107 are located in the 3′ UTR of *AVPR1A*. TargetScan predicted that the miR-526b target site is located at rs11174815, and the miR-375 and miR-186 target sites are located at rs3803107. Plasma levels of miR-526b, miR-375, and miR-186 were higher in hypertensive patients than in healthy subjects (*P*<0.001). The data in the present study are not sufficient to explain the increase in the plasma levels of miRs in hypertensive patients, which may be due to a combination of factors, such as long-chain ncRNA expression regulation, which is an interesting research direction. Therefore, we speculate that the regulation of *AVPR1A* expression by miR-526b, miR-375, and miR-186 may be related to the occurrence of hypertension. In order to confirm our hypothesis, we used CRISPR/Cas9 technology to construct HUVECs with CC or CA/AA genotypes at rs11174811 and HUVECs with GG or GA/AA genotypes at rs3803107. After transfection of miR-526b, miR-375, and miR-186 mimics, *AVPR1A* mRNA expression level increased, indicating that these miRNAs can regulate the expression of *AVPR1A*, which was consistent with the results in hypertensive patients. Analysis of the expression levels from different genotypes in transfected cells showed that the expression level of *AVPR1A* mRNA in the mutant cells was significantly higher than that in the wild-type cells. The results of western blotting showed that AVPR1A protein expression was also significantly higher in the mutant cells than in wild-type cells. From these results, it can be concluded that the rs11174811 and rs3803107 SNPs affect the regulation of *AVPR1A* expression by miRNAs, which may be related to the effect of sequence variation on the binding efficiency of miRNAs to their target sites. Human genes can be regulated by a variety of miRNAs [22,23] and genetic variation at miRNA target sites in the 3′ UTR of genes affects miRNA target recognition [24]. In addition, in the present study, the plasma levels miR-526b, miR-375, and miR-186 were not correlated with the genotypes at rs11174811 and rs3803107 (*P*>0.05). This suggests that miRNA target recognition efficiency is key to determining the expression level of *AVPR1A* in the presence of the same level of miRNA. Although we do not have direct evidence to support the inhibition of *AVPR1A* expression by miRNA via binding to *AVPR1A* mRNA, it may also have involved other mechanisms and more direct evidence is needed. The expression of *AVPR1A* was not as expected when transfected with 100 ng of ssEGFP miRNA *in vitro*, and there may be other unknown reasons, but the difference in *AVPR1A* expression levels between the different genotypes was consistent with our expectations.

There are some shortcomings in the present study. First, the sample size was insufficient, the number of homozygotes was small, and the analysis error was large. Therefore, we combined heterozygotes and homozygotes in the analysis. Second, there may be some differences between the *in vitro* cell model used here and the regulation of miRNA expression *in vivo*. In addition, the occurrence of hypertension may involve a complex effect of multiple genes and non-genetic factors. The role of a single gene may be relatively minor and further research is needed.

## Conclusion

rs11174811 and rs3803107 SNPs in the *AVPR1A* gene are associated with the risk of hypertension in the Chinese Han population. The mechanism may be that variation at rs11174811 affects the regulation of *AVPR1A* expression via miR-526b, and variation at rs3803107 affects the regulation of *AVPR1A* expression via miR-375 and miR-186.

## Abbreviations

AVP: arginine vasopressin
BMI: body mass index
CF: myocardial fibroblast
DBP: diastolic blood pressure
HRP: horseradish peroxidase
HUVEC: human umbilical vein endothelial cell
HWE: Hardy-Weinberg equilibrium
MAF: minimum allele frequency
OR: odds ratio
SBP: systolic blood pressure
SNP: single nucleotide polymorphism
